# Asymptomatic SARS‐CoV‐2 Infection: Association Involving the HLA‐B*15 Allele Group in Brazilian Individuals

**DOI:** 10.1111/tan.70262

**Published:** 2025-06-01

**Authors:** Christiane Maria Ayo, Henrique Magalhães, Marcos Paulo Miola, Cinara Cássia Brandão, Maurício Lacerda Nogueira, Cássia Fernanda Estofolete, Victor Hugo de Souza, Jeane Eliete Laguila Visentainer, Luiz Carlos de Mattos

**Affiliations:** ^1^ Departamento de Doenças Dermatológicas, Infecciosas e Parasitárias, Laboratório de Imunogenética Faculdade de Medicina de São José do Rio Preto (FAMERP) São José do Rio Preto São Paulo Brazil; ^2^ Departamento de Doenças Dermatológicas, Infecciosas e Parasitárias, Laboratório de Pesquisa Em Virologia Faculdade de Medicina de São José do Rio Preto (FAMERP) São José do Rio Preto São Paulo Brazil; ^3^ Departamento de Ciências Básicas da Saúde, Laboratório de Imunogenética Universidade Estadual de Maringá (UEM) Maringá Paraná Brazil

**Keywords:** asymptomatic, COVID‐19, *HLA* Class I, SARS‐CoV‐2

## Abstract

The aim of this study was to investigate the influence of *HLA‐A*, *‐B* and *‐C* polymorphisms on the clinical course of SARS‐CoV‐2 infection and on the progression of COVID‐19 in a population from southeastern Brazil. This study included 478 unvaccinated individuals. Of these, 369 were hospitalised with critical/severe (*n* = 309) or moderate/mild (*n* = 60) symptoms, and 109 were asymptomatic. The control group consisted of 150 volunteer bone marrow donors, recruited in the pre‐pandemic period. The HLA‐B*15 allele group (adjusted *p* value < 0.001) was associated with a protective factor against symptomatic infection. Investigating the effects of the distribution of *HLA* alleles on susceptibility and resistance to SARS‐CoV‐2 may provide a better understanding of the clinical course of infection in different geographical regions.

## 1 Introduction

Despite the high number of individuals infected with SARS‐CoV‐2 who develop varying degrees of clinical severity of COVID‐19, approximately 20% of infections are asymptomatic [1]. It remains unclear why some individuals remain asymptomatic or clear the infection without complications, while others develop severe disease, even in the absence of known associated risk factors [2].

Host genetics have been proposed as a key factor in the different immune responses to infection and disease progression. In fact, the *HLA* system is crucial in directing antiviral immunity, since *HLA* gene polymorphisms determine the presentation efficiency of SARS‐CoV‐2 epitopes to CD4^+^ and CD8^+^ T cells by HLA Class I (*HLA‐I*) and HLA Class II (*HLA‐II*) molecules, respectively [3]. Thus, the genetic diversity of *HLA* alleles across different populations may influence COVID‐19 severity by shaping the presentation of viral antigens to the immune system [4].

In particular, HLA‐I molecules play a key role in directing effective immune response during SARS‐CoV‐2 infections. It was demonstrated that many SARS‐CoV‐2 epitopes for CD8^+^ T cells are presented in an HLA‐restricted manner [5], and early development of the cytotoxic CD8^+^ T cell response is correlated with effective viral clearance and mild disease [3]. The HLA‐B*46:01 allele was associated with SARS risk [6]; corroborating this result, in silico analyses have pointed to this allele presenting low predicted binding to peptides of SARS‐CoV‐2, suggesting susceptibility to COVID‐19 [5].

Several studies of genetic association have investigated the role of *HLA* genes in SARS‐CoV‐2 infection and its clinical progression [7, 8]. The diverse results observed in different populations indicate that different *HLA* haplotypes and alleles may directly or indirectly affect the ability of some antigens to bind to HLA molecules. *HLA* alleles are variable, especially in the highly admixed Brazilian population [9]. Hence, investigating the effects of the distribution of *HLA* alleles on susceptibility or resistance to SARS‐CoV‐2 may help to better understand the clinical course of infection in different geographical regions [7]. The aim of this study was to investigate the influence of *HLA‐A*, *‐B* and *‐C* polymorphisms on the clinical course of SARS‐CoV‐2 infection and the progression of COVID‐19 in a population from southeastern Brazil.

## 2 Materials and Methods

### 2.1 Subject Selection

This study included 478 unvaccinated individuals with a positive diagnosis for SARS‐CoV‐2 during the 2020–2021 pandemic. Of these, 369 were hospitalised in Hospital de Base of the Fundação Faculdade Regional de Medicina (HB‐FUNFARME) in São José do Rio Preto, SP, Brazil due to critical/severe (*n* = 309) or moderate/mild (*n* = 60) symptoms of COVID‐19. The other 109 were asymptomatic individuals living in the same geographic region. The control group for the allele group frequency analyses consisted of 150 volunteer bone marrow donors, eligible for donation, recruited at the blood bank in São José do Rio Preto in the pre‐pandemic period.

Inclusion criteria were a positive diagnosis of SARS‐CoV‐2 based on clinical evaluation and laboratory diagnosis, and no familial relationship to other study participants.

Due to the historically admixed nature of the Brazilian population, study participants were considered to have diverse ancestral backgrounds, although they self‐reported themselves as ‘European descent’ and ‘No European descent’ (African descent or mixed African with European ancestry). The potential impact of ancestral background on allele frequency variations was minimised by matching individuals in groups with similar ancestries.

This study was approved by the Research Ethics Committee of the da Medical School in São José do Rio Preto (CAAE 32293014.0.0000.5415). An informed consent form was signed by all participants.

### 2.2 Clinical Characterisation of Patients

SARS‐CoV‐2‐infected individuals were classified according to clinical form (asymptomatic or symptomatic) and severity of illness (clinical symptoms) as recommended by the World Health Organization (WHO, available at: https://iris.who.int/handle/10665/332196): (a) Asymptomatic—no presence of any symptoms consistent with COVID‐19; (b) mild illness—the presence of the most common symptoms such as fever, cough, dysfunction of smell (anosmia) and taste (dysgeusia) but no shortness of breath (dyspnea); (c) moderate illness—the presence of the most common symptoms including dyspnea and clinical or radiographic evidence of lower respiratory tract disease but no hypoxemia (blood oxygen saturation ≥ 94%) in which hospitalisation may be required; (d) severe illness—the presence of the most common symptoms, dyspnea, pulmonary impairment, hypoxemia (blood oxygen saturation < 90%) and hospitalisation in an intensive care unit (ICU) and (e) critical illness—respiratory failure (acute respiratory distress syndrome), multiple organ dysfunction or septic shock.

The symptomatic patients (*n* = 369) with mild (7.0%), moderate (9.2%), severe (9.8%) or critical (74.0%) symptoms of COVID‐19 were grouped into two subgroups for the purpose of genetic comparisons: Patients that presented mild or moderate symptoms, defined as those who showed no hypoxemia, were grouped into M group; deceased and/or hospitalised in ICU requiring ventilation support (those with severe or critical symptoms) were grouped into SC group. For these patients, the SARS‐CoV‐2 infection was confirmed by real‐time quantitative reverse transcription‐polymerase chain reaction (RT‐qPCR). Additionally, they were tested using a viral respiratory panel (influenza A, influenza B and respiratory syncytial virus) to exclude similar pathogens and coinfections.

The asymptomatic individuals were recruited from a prospective cohort study for SARS‐Cov‐2 infection surveillance carried out in a restricted neighbourhood in the municipality of São José do Rio Preto. Individuals who presented IgG anti‐SARS‐CoV‐2 antibodies by serological tests were invited for an interview carried out by doctors collaborating on this study to clarify and confirm whether or not they presented symptoms suggestive of COVID‐19 in 2020 and 2021. Subjects who reported the absence of symptoms were included in the group of asymptomatic individuals (*n* = 109). Based on peripheral blood tests, it can be assumed that individuals who presented anti‐SARS‐CoV‐2 antibodies had been exposed to this virus, infected, and for this had developed a humoral adaptive immune response. The option for a serological test arose from the recognition that asymptomatic individuals often lack symptoms suggestive of COVID‐19 or other respiratory viral diseases. Consequently, they may not seek medical attention and, therefore, remain untested by molecular methods like qPCR.

Standardised data were collected on demographic features, ethnicity, presence of comorbidities (hypertension and other cardiovascular diseases, diabetes, asthma, pulmonary disease, chronic kidney disease, immunosuppression, neurologic diseases, chromosomal diseases, haematological diseases, liver disease, cancer and obesity), recent pregnancy and clinical symptoms (fever, cough, sore throat, dyspnea, low saturation, diarrhoea, vomiting, fatigue, abdominal pain, respiratory distress, altered taste and anosmia) during the admission at a health centre (symptomatic) or during an interview (asymptomatic).

### 2.3 Detection of the SARS‐CoV‐2 Infection

The viral RNA was extracted using the QIAmp Viral RNA Mini Kit (Qiagen, Hilden, Germany) and detected by TaqMan‐based Real‐Time PCR (Promega, Madison, USA) using a Centers for Disease Control (CDC, USA) protocol with two primer/probe sets which amplify the virus nucleocapsid (N) gene (2019‐nCoV_N1 and 2019‐nCoV_N2). The Human RNase P (RP) primer/probe set was included to detect the gene in a control sample [10].

The enzyme‐linked immunosorbent assay (ELISA) methodology was used for the qualitative determination of IgG levels against the nucleocapsid (N) and spike (S) proteins of SARS‐CoV‐2 (CT‐Vacinas, Brazil) [11]. Positive and negative controls were included in all reactions, and the samples were tested in duplicate.

### 2.4 HLA Genotyping

*HLA‐I* (*‐A*, *‐B* and *‐C*) were genotyped by polymerase chain reaction‐sequence specific oligonucleotide probe (PCR‐SSO) protocols with Luminex technology using the LABType CWD kit (One Lambda Inc., Canoga Park, CA, USA) with low/medium resolution according to the manufacturer's instructions. Hybridization was verified by fluorescence intensity using flow cytometry (FLEXMAP 3D Instrument Systems). The results were interpreted using *HLA* FUSION software, version 4.6 (One Lambda Inc., Canoga Park, CA, USA).

### 2.5 Statistical Analysis

The Arlequin software version 3.5.1.3 (available at: https://cmpg.unibe.ch/software/arlequin3) was used to calculate the *HLA‐I* allele group frequencies and confirm Hardy–Weinberg equilibrium. The *t* test was used to compare continuous variables between the groups. The chi‐square test (*χ* ^2^) was used to determine *p* values and odds ratios (OR) along with 95% confidence intervals (95% CIs) when categorical data were compared between the groups. Statistical analyses were performed using the R programming language version 4.1.2 (available at: https://www.r‐project.org/). The univariate *p* values and OR were adjusted accordingly to age (≥ 60 years), sex (male) and presence of at least one risk factor such as underlying diseases to determine the independence of genetic variables using a multivariate logistic regression analysis available. In addition, the process automation package such as ‘dplyr’ also used. The multivariate *p* values were corrected for multiple comparisons using the Bonferroni model (adjusted *p* value). Adjusted *p* values < 0.05 were considered statistically significant.

## 3 Results

### 3.1 General Characteristics and Comorbidity of Participants, and Clinical Characteristics of the Hospitalised COVID‐19 Patients

The general and clinical characteristics of the participants are shown in Table S1. Only significant results are shown in Table 1. Of the 369 symptomatic patients, 84.5% (*n* = 312) had one or more comorbidities, 57.2% (*n* = 211) died over the course of COVID‐19 whereas 42.8% (*n* = 158) recovered from the disease; 83.8% (*n* = 309) had severe/critical COVID‐19 (SC group) and 16.2% (*n* = 60) presented mild/moderate COVID‐19 (M group). Neurological disorders, hypertension, other cardiovascular disorders and obesity were more common in the SC group with frequencies of 86.7%, 46.3%, 59.2% and 35.2%, respectively. These comorbidities were risk factors for severe/critical COVID‐19 (*p* value < 0.05). These same comorbidities were more frequent in symptomatic (74.0%, 41.5%, 53.4% and 74.5%, respectively) than in asymptomatic individuals (0.9%, 15.6%, 1.8% and 0.9%, respectively) and thus were associated with a greater chance of developing symptomatic SARS‐Cov‐2 infection (*p* value < 0.05). Of the 120 individuals with asymptomatic infections, 74.3% reported no known COVID‐19‐associated comorbidity.

**TABLE 1:** Differences on general characteristics and comorbidity of SARS‐CoV‐2‐infected participants, and clinical characteristics of the symptomatic COVID‐19 patients.

Characteristics	SARS‐CoV‐2‐infected participants *n* (%)		Comparisons, p^a^; OR (95% CI)
	Asymptomatic (*n* = 109)	Symptomatic (*n* = 369)	
Sex			
Male	39 (35.8)	191 (51.8)	0.004; 1.92 (1.23, 3.01)
Female	70 (64.2)	178 (48.2)	
Mean age ± SD	36.15 ± 17.7	56.82 ± 17.39	< 0.0001; t = 10.85
Hypertension	17 (15.6)	153 (41.5)	0.000001; 3.82 (2.23, 6.84)
Others cardiovascular disorder	2 (1.8)	197 (53.4)	**<** 0.0000001; 40.08 (11.67, 244.60)
Diabetes	5 (4.6)	136 (36.8)	< 0.0000001; 16.27 (6.89, 46.36)
Neurological disorder	1 (0.9)	273 (74.0)	**<** 0.0000001; 791.1 (150.9, 16550.0)
Pneumopathy	0 (0.0)	8 (2.1)	0.004; undefined (3.36, undefined)
Kidney disorder	0 (0.0)	25 (6.8)	0.009; undefined (2.95, undefined)
Cancer	0 (0.0)	20 (5.4)	0.02; undefined (2.28, undefined)
	**SC group (*n* = 309)**	**M group (*n* = 60)**	
Mean age ± SD	53.91 ± 17.87	60.0 ± 17.39	0.01; t = ‐2.46
Diarrhea	29 (9.4)	13 (21.6)	0.01; 0.37 (0.18, 0.79)
Low blood oxygen saturation	276 (89.3)	36 (60.0)	< 0.0001; 5.53 (2.93, 10.45)
Dyspnea	273 (88.3)	14 (23.3)	< 0.0001; 24.54 (12.4, 50.43)
Respiratory distress	193 (62.5)	18 (30.0)	< 0.0001; 3.86 (2.14, 7.17)
Hypertension	143 (46.3)	10 (16.7)	00003; 4.29 (2.15, 9.20)
Others cardiovascular disorder	183 (59.2)	14 (23.3)	0000003; 4.47 (2.54, 9.28)
Neurological disorder	268 (86.7)	5 (8.3)	< 0.0001; 70.5 (28.2, 209.6)
Obesity	109 (35.2)	10 (16.7)	0.007; 2.71 (1.35, 5.84)
Ventilator support	307 (99.3)	25 (41.6)	< 0.0001; 207.7 (54.81, 1348)
ICU care required	303 (98.0)	0 (0.0)	< 0.0001; undefined (609.2, undefined)
Death	211 (68.3)	0 (0.0)	< 0.0001; undefined (34.51, undefined)

*Note:* Only significant results are shown.

Abbreviations: M, mild/moderate; SC, severe/critical.

^a^ *p*‐value: calculated using the t test ou χ2.

Multiple clinical characteristics were analysed in the severe course of COVID‐19. Oxygen saturation was significantly different between the COVID‐19 subgroups (*p* value < 0.0001). As expected, the mean blood oxygen saturation was significantly lower in people with severe/critical COVID‐19. Moreover, the percentage of subjects with respiratory distress (*p* value < 0.0001) and dyspnea (*p* value < 0.0001) was higher in the SC Group compared to the M group. Treatment with ventilator support, intensive care requirements, and death were also significantly higher in the SC group than in the M group (*p* value < 0.0001).

The mean age of symptomatic patients (56.82 ± 17.39 years) was significantly higher than the mean age of the group of individuals with asymptomatic infections (36.15 ± 17.7 years—*p* value < 0.0001). Differences in age were also observed between the subgroups of patients: those with mild/moderate COVID‐19 (mean: 60.0 ± 17.39 years) were older than those who had severe/critical disease (mean: 53.91 ± 17.87 years—*p* value = 0.01). However, the SC and M groups presented the same median age (60 years).

The distribution according to sexes was homogeneous within symptomatic patients, with 51.8% (*n* = 191) males and 48.2% (*n* = 178) females. Furthermore, no significant differences were observed between the M and SC Groups. However, males more often had symptomatic than asymptomatic infections (51.8% vs. 35.8%, *p* value = 0.004). Asymptomatic infections were predominant in females (*p* = 0.00004; OR = 0.31; CI = 0.17, 0.54).

### 3.2 Comparison of HLA Class I Frequencies of SARS‐CoV‐2 Infected Individuals With Different Clinical Outcomes and Control Subjects

In the control group, the *HLA‐I* frequencies were in Hardy–Weinberg equilibrium (*p* > 0.05).

Table 2 presents, only for the significant differences, the distribution of *HLA‐A*, *‐B* and *‐C* allele group frequencies of infected patients (symptomatic and asymptomatic) and non‐infected individuals, as well as the SC and M groups. The B*15 allele group was more frequent in the asymptomatic patient group than in the symptomatic patient group (*p* = 0.00001) and the Control Group (*p* value = 0.02). The B*44 allele group was found to be more frequent in symptomatic patients compared with asymptomatic patients (*p* value = 0.02). To verify which allele groups contributed to a worse clinical outcome of COVID‐19, comparisons were made between the SC and M groups: The HLA‐C*04 allele group was more frequent (*p* value = 0.001) and the HLA‐B*07, HLA‐B*13, HLA‐C*07 and HLA‐C*16 allele groups were less frequent (*p* = 0.03, *p* = 0.01, *p* = 0.02 and *p* = 0.02, respectively) in patients with severe/critical COVID‐19 (Table S2).

**TABLE 2:** Association of *HLA* Class I allele group and SARS‐CoV‐2 susceptibility and severity of COVID‐19.

Allele groups	SARS‐CoV‐2‐infected and controls participants *n* (%)		*p*; OR (95% CI)	Adjusted *p*; OR (95% CI)
HLA‐B*07	SC group (*n* = 309)	M group (*n* = 60)		
	22 (3.6)	10 (8.3)	0.03; 0.40 (0.18, 0.91)	
HLA‐B*13	SC group (*n* = 309)	M group (*n* = 60)		
	10 (1.6)	7 (5.8)	0.01; 0.26 (0.09, 0.75)	
HLA‐B*15	Symptomatic (*n* = 369)	Asymptomatic (*n* = 109)		
	51 (6.9)	37 (17.0)	< 0.00001; 0.36 (0.21, 0.57)	< 0.001; 0.33 (0.18, 0.60)
	Asymptomatic (*n* = 109)	Control (*n* = 150)		
	37 (17.0)	25 (8.3)	0.004; 0.44 (0.25,0.76)	
HLA‐B*44	Symptomatic (*n* = 369)	Asymptomatic (*n* = 109)		
	76 (10.3)	11 (5.0)	0.02; 2.15 (1.15, 0.57)	
HLA‐C*04	SC group (*n* = 309)	M group (*n* = 60)		
	127 (20.6)	9 (7.5)	0.001; 3.18 (1.62, 6.85)	
HLA‐C*07	SC group (*n* = 309)	M group (*n* = 60)		
	114 (18.4)	34 (28.3)	0.02; 0.44 (0.23, 0.87)	
HLA‐C*16	SC group (*n* = 309)	M group (*n* = 60)		
	34 (5.5)	14 (11.4)	0.02; 0.44 (0.23, 0.87)	

*Note: p*‐value: calculated using the χ2. Adj. *p*‐value: refers to the logistic regression analysis adjusted for age, sex, and presence of at least one risk factor and corrected by Bonferroni. Only significant results are shown.

Abbreviations: M, moderate/mild; SC, severe/critical.

### 3.3 Association of HLA Allele Groups and SARS‐CoV‐2 Susceptibility and Severity

After multivariate analysis, a significant difference was found for the HLA‐B*15 allele group (adjusted *p* value < 0.001) as a protective factor against symptomatic infection. None of the HLA‐I allele groups increased the risk of severe disease in SARS‐CoV‐2 infected patients (Table 2).

## 4 Discussion

The wide spectrum of COVID‐19 clinical presentations within different populations, ranging from mild‐to‐severe symptomatic infections and also asymptomatic cases, reflects the variable capacity of host immune responses to control the virus [5]. Many studies are investigating genetic factors that could influence the clinical course of SARS‐CoV‐2. Several studies have focused on the variability of classical *HLA* genes, as they play a crucial role in the antigen presentation pathway and can confer differential susceptibility and severity of diseases [3]. Of course, in addition to the genetic factors, advanced age, presence of certain comorbidities, lifestyle habits, social and environmental conditions, also influence susceptibility to SARS‐CoV‐2 infection and the severity of COVID‐19 [12].

The results of this study confirmed previously reported clinical parameters and comorbidities as risk factors associated with severe COVID‐19 [12, 13]. According to demographic parameters, although the group of patients with severe/critical COVID‐19 had a lower mean age than those with mild/moderate disease, the median of these two groups was the same, indicating that the presence of children in the SC group influenced the mean age, and therefore contradicted the various data published in the literature that older age is associated with the severity of COVID‐19 disease progression and mortality [13]. It is also pertinent to consider the immaturity of the immune system in children as a potential risk factor for severe disease [14]. Furthermore, asymptomatic infections were more frequently observed in females, a finding consistent with prior reports indicating a higher likelihood of asymptomatic COVID‐19 in women [15]. Due to hormonal factors, men are predisposed to being more susceptible to symptomatic COVID‐19 as testosterone fosters viral entry into host cells and the systemic dissemination of SARS‐CoV‐2 [13].

At the population level, highly polymorphic genetic variants are likely involved in conferring immunity against emerging infectious diseases. Polymorphisms of *HLA* genes differ between populations worldwide. Brazil, in particular, has a highly genetically diverse population. The admixture history, which varies across Brazilian geographic regions, is likely a key factor influencing the observed variation in *HLA* diversity throughout the country [9]. Overall, Brazilian population *HLA‐I* data shows that at locus A, the most common allele is HLA‐A*01:02 (21.04%), followed by HLA‐A*24:02 (8.41%) and HLA‐A*01:01 (8.85%). At Locus B, the most common alleles are HLA‐B*07:02 (6.22%), HLA‐B*35:01 (5.39%) and HLA‐B*08:01 (4.65%) and at Locus C, HLA‐C*04:01 (16.55%), HLA‐C*07:01 (11.98%) and HLA‐C*07:02 (6.32%) [16].

In this study, a positive correlation was found between the HLA‐B*15 allele group and asymptomatic SARS‐CoV‐2 infection (17.0%—asymptomatic vs 9.2%—symptomatic). HLA‐I molecules perform a function in the presentation of endogenous pathogenic agents to CD8^+^ T cells, with the interaction between SARS‐CoV‐2 peptides and presenting molecules characterised by varying degrees of binding affinity, allowing for the categorisation of these interactions: strong, regular, weak and non‐binder [17]. Molecules encoded by the HLA‐B*15 lineage appear to be the best binders correlating with progression of SARS‐CoV‐2 infection. The HLA‐B*15:03 allele has been predicted to exhibit high binding affinity for viral peptides, including SARS‐CoV‐2 peptides [5, 17], with its presence having been associated with protection against COVID‐19 [5]. The HLA‐B*15:03 allele is more frequent in populations of sub‐Saharan Africa, and maybe, in proportion, this allele is the reason for low mortality on this continent due to its effective presentation ability [4]. The other strong binders of this lineage, including HLA‐B*15:17, ‐B*15:25, ‐B*15:39 and ‐B*35:10 [5], are very uncommon in populations worldwide [4]. Thus, the frequencies of strong and weak HLA binders vary depending on the geographical region [8].

A recently published article showed a strong association between the HLA‐B*15:01 allele and asymptomatic SARS‐CoV‐2 infection in two independent cohorts with European ancestry [18]. The authors found an association between HLA‐B*15:01 and asymptomatic SARS‐CoV‐2 infection, noting that individuals with this allele were twice as likely to remain asymptomatic after infection. Furthermore, the HLA‐B*15 allele group was found to be highly expressed on the surface of human platelets; at the allelic level, most of the individuals with HLA‐B*15 had HLA‐B*15:01 [19]. Platelets have several immune functions, and so the higher expression of HLA‐B*15:01 could enhance activation of the immune response, providing viral clearance leading to asymptomatic COVID‐19 among individuals with SARS‐CoV‐2 infections [20].

In contrast, a lack of association of classical *HLA* alleles, including HLA‐B*15:01, with asymptomatic SARS‐CoV‐2 infections was reported in both North American [21] and Spanish [22] populations. It is known that the frequency of HLA‐B*15:01 exhibits variations at the continental level, within American populations, and even among European countries [9]. The inconsistency observed between studies may be partially caused by the influence of ethnicity on *HLA* allele distributions. The observed variation in allele frequencies across geographic regions raises the possibility that protective *HLA* alleles identified in one population [8] may also be important in others. This, in turn, suggests that either other *HLA* alleles contribute to protection against infection, or environmental factors play a significant role in disease progression. Differences in research methods, sample size and selection of subjects may also produce diverging results.

One limitation of this study is that no distinction was made between viral strains in SARS‐CoV‐2‐infected individuals. During a pandemic period with sustained transmission are expected viral variants over time across different geographic regions and between populations [23]. The predominant SARS‐CoV‐2 variants circulating in the southeastern region of Brazil during the study period were dominated by lineages B.1.1.28 and B.1.1.33 from February 2020 to November 2020. The P.1 (Gamma) became the dominant variant by the beginning of 2021 and was rapidly outcompeted by B.1.617.2 (Delta) from June 2021. Certain variants have mutations that may impact transmission, immune escape and virulence, influencing clinical outcomes [24]. Another limitation is that the absence of all symptoms in the asymptomatic group was self‐reported. Although we cannot definitively state that these individuals were entirely free of any symptoms, asymptomatic disease was clinician‐defined and the data found showed a very robust genetic association, corroborating with the literature on the HLA‐B*15 lineage and the progression of SARS‐CoV‐2 infection [5, 17, 18, 20]. Further studies are needed, such as CD8^+^ T cell cytotoxicity assay and cytokine analysis to assess immune responses in HLA‐B*15 allele group carriers. Studies involving platelet functions also would provide valuable insights to support the findings of this study.

*HLA‐I* genes have an important function in CD8^+^ T cell repertoire selection. Variations due to the polymorphisms could have a positive effect on the host immune response infected by SARS‐CoV‐2, since certain alleles may enhance viral clearance and host immune response leading to asymptomatic infection [4]. Based on the results published in different populations, it seems that different alleles in each play a role in the exacerbation and occurrence of the disease. In conclusion, the results obtained herein suggest that the HLA‐B*15 allele group is a protective factor against symptomatic infection in a population from southeastern Brazil. HLA‐B*15 can be considered a research target to be further investigated in Brazilian individuals infected with SARS‐CoV‐2.

## Author Contributions

L.C.M., C.M.A., J.E.L.V. and C.C.B. conceived and designed the experiments. C.M.A. performed the molecular experiments and analysed the data. C.M.A. and V.H.S. performed the statistical analysis. C.M.A. and L.C.M. wrote the paper. H.M., M.P.M. and C.F.E. performed triage on hospitalised patients. C.F.E. performed the inclusion of participants and developed the clinical evaluation. C.F.E. and M.L.N. contributed to the laboratory diagnosis of SARS‐CoV‐2. All authors read and approved the manuscript.

## Conflicts of Interest

The authors declare no conflicts of interest.

## Supporting information


**Table S1.** Supporting Information.

**Table S2.** Supporting Information.

## Data Availability

The data that support the findings of this study are available from the corresponding author upon reasonable request.
