# Supplementary material for: Asymptomatic SARS‐CoV‐2 Infection: Association Involving the HLA‐B*15 Allele Group in Brazilian Individuals
Source: HLA. 2025 Jun 1;105(6):e70262. doi: 10.1111/tan.70262 (PMC12127060; doi:10.1111/tan.70262)
Supplement: Supplementary file 1 — Table S1. Supporting Information. [file TAN-105-e70262-s002.pdf]

Table S1: General characteristics and comorbidity of participants, and clinical characteristics of the hospitalized COVID-19 patients.

Characteristics	Asymptomatic n = 109	Hospitalized COVID-19 patients<br>Symptomatic n = 369	SC group n = 309	M group n = 60
**Sex**	**n (%)**	**n (%)**	**n (%)**	**n (%)**
Male	39 (35.8)	191 (51.8)	161 (52.1)	30 (50.0)
Female	70 (64.2)	178 (48.2)	148 (47.9)	30 (50.0)
**Age (years)**				
Mean ± SD	36.15 ± 17.7	56.82 ± 17.39	53.91 ± 17.87	60.0 ± 17.39
Median (Range)	36 (6-83)	60 (1-90)	60 (1-90)	60 (30-90)
**Ancestry Backgrounds**				
European descent	69 (63.3)	261 (70.7)	223 (72.2)	41 (68.3)
No European descent	40 (36.7)	108 (29.3)	86 (27.8)	19 (31.7)
**Clinical characteristics**				
Fever	-		166 (53.7)	40 (66.6)
Cough	-		200 (64.7)	40 (66.6)
Sore throat	-		43 (13.9)	12 (20.0)
Diarrhea	-		29 (9.4)	13 (21.6)
Vomiting	-		24 (7.8)	5 (8.3)
Abdominal pain	-		96 (31.1)	8 (13.3)
Low blood oxygen saturation	-		276 (89.3)	36 (60.0)
Dyspnea	-		273 (88.3)	14 (23.3)
Respiratory distress	-		193 (62.5)	18 (30.0)
Fatigue	-		49 (15.8)	12 (20.0)
Anosmia and/or hyposmia	-		115 (37.2)	26 (43.3)
**Comorbidity (one or more)**				
Hypertension	17 (15.6)	153 (41.5)	143 (46.3)	10 (16.7)
Others cardiovascular disorder	2 (1.8)	197 (53.4)	183 (59.2)	14 (23.3)
Hematological disorder	0 (0.0)	11 (3.0)	9 (2.9)	2 (3.3)
Liver disorder	0 (0.0)	15 (4.1)	10 (3.2)	5 (8.3)
Asthma	2 (1.8)	9 (2.4)	8 (2.6)	1 (1.6)
Diabetes	5 (4.6)	136 (36.8)	117 (37.9)	19 (31.6)
Neurological disorder	1 (0.9)	273 (74.0)	268 (86.7)	5 (8.3)
Pneumopathy	0 (0.0)	8 (2.1)	6 (1.9)	2 (3.3)
Immunocompromised status	0 (0.0)	28 (7.6)	26 (8.4)	2 (3.33)

Kidney disorder	0 (0.0)	25 (6.8)	20 (6.5)	5 (8.3)
Obesity	NI	119 (32.2)	109 (35.2)	10 (16.7)
Cancer	0 (0.0)	20 (5.4)	17 (5.5)	3 (5.0)
Recent childbirth	0 (0.0)	1 (0.3)	1 (0.3)	0 (0.0)
Chromosomal disease	0 (0.0)	1 (0.3)	1 (0.3)	0 (0.0)
**Others**				
Ventilator support	-	332 (90.0)	307 (99.3)	25 (41.6)
ICU care required	-	303 (82.1)	303 (98.0)	0 (0.0)
Death	-	211 (57.2)	211 (68.3)	0 (0.0)

SC: severe/critical; M: mild/moderate.
