# Supplementary material for: Asymptomatic SARS‐CoV‐2 Infection: Association Involving the HLA‐B*15 Allele Group in Brazilian Individuals
Source: HLA. 2025 Jun 1;105(6):e70262. doi: 10.1111/tan.70262 (PMC12127060; doi:10.1111/tan.70262)
Supplement: Supplementary file 2 — Table S2. Supporting Information. [file TAN-105-e70262-s001.pdf]

Table S2: Distribution of *HLA* allele group frequencies in SARS-CoV-2 infection susceptibility and severity and in control individuals.

				Hospitalized COVID-19 patients		
Alelle groups	Control<br>n = 150	Total SARS-CoV-2<br>n = 478	Asymptomatic<br>n = 109	Symptomatic<br>n = 369	SC group<br>n = 309	M group<br>n = 60
HLA-A						
***01**	21 (7.0)	77 (8.1)	18 (8.3)	59 (8.0)	54 (8.7)	5 (4.2)
***02**	86 (28.7)	240 (25.1)	54 (24.8)	186 (25.2)	161 (26.1)	25 (20.8)
***03**	25 (8.3)	86 (9.0)	17 (7.8)	69 (9.3)	55 (8.9)	14 (11.7)
***11**	13 (4.3)	39 (4.1)	11 (5.0)	28 (3.8)	25 (4.0)	3 (2.5)
***23**	13 (4.3)	45 (4.7)	12 (5.5)	33 (4.5)	29 (4.7)	4 (3.3)
***24**	31 (10.3)	102 (10.7)	24 (11.5)	78 (10.6)	67 (10.8)	11 (9.2)
***25**	7 (2.3)	19 (2.0)	4 (1.8)	15 (2.0)	12 (1.9)	3 (2.5)
***26**	16 (5.3)	40 (4.2)	7 (3.2)	33 (4.5)	28 (4.5)	5 (4.2)
***29**	15 (5.0)	53 (5.5)	12 (5.5)	41 (5.6)	33 (5.3)	8 (4.2)
***30**	15 (5.0)	53 (5.5)	15 (6.9)	38 (5.1)	31 (5.0)	7 (5.8)
***31**	8 (2.7)	36 (3.8)	7 (3.2)	29 (3.9)	23 (3.7)	6 (5.0)
***32**	13 (4.3)	34 (3.6)	7 (3.2)	27 (3.7)	21 (3.4)	6 (5.0)
***33**	7 (2.3)	25 (2.6)	5 (2.3)	20 (2.7)	15 (2.4)	5 (4.2)
***34**	4 (1.3)	16 (1.7)	4 (1.8)	12 (1.6)	9 (1.5)	3 (2.5)
***36**	3 (1.0)	8 (0.8)	2 (0.9)	6 (0.8)	6 (1.0)	0 (0.0)
***66**	0 (0.0)	2 (0.2)	0 (0.0)	2 (0.3)	1 (0.2)	1 (0.8)
***68**	15 (5.0)	56 (5.9)	11 (5.0)	45 (5.1)	34 (5.5)	11 (9.2)
***69**	3 (1.0)	8 (0.8)	3 (1.4)	5 (0.7)	3 (0.5)	2 (1.7)
***74**	5 (1.7)	14 (1.5)	4 (1.8)	10 (1.4)	9 (1.5)	1 (0.8)
***80**	0 (0.0)	2 (0.2)	0 (0.0)	2 (0.3)	2 (0.3)	0 (0.0)
HLA-B						
***07**	19 (6.3)	41 (4.3)	9 (4.1)	32 (4.3)	22 (3.6)	10 (8.3)
***08**	13 (4.3)	42 (4.4)	11 (5.0)	31 (4.2)	23 (3.7)	8 (6.7)
***13**	7 (2.3)	22 (2.3)	5 (2.3)	17 (2.3)	10 (1.6)	7 (5.8)
***14**	10 (3.3)	72 (7.5)	10 (4.6)	62 (8.4)	48 (7.8)	14 (11.7)
***15**	25 (8.3)	88 (9.2)	37 (17.0)	51 (6.9)	44 (7.1)	7 (5.8)
***18**	23 (7.7)	55 (5.8)	15 (6.9)	40 (5.4)	35 (5.7)	5 (4.2)
***27**	5 (1.7)	16 (1.7)	1 (0.5)	15 (2.0)	14 (2.3)	1 (0.8)
***35**	44 (14.7)	142 (14.9)	32 (14.7)	110 (14.9)	95 (15.4)	15 (12.5)
***37**	8 (2.7)	11 (1.2)	1 (0.5)	10 (1.4)	10 (1.6)	0 (0.0)
***38**	6 (2.0)	20 (2.1)	3 (1.4)	17 (2.3)	15 (2.4)	2 (1.7)
***39**	7 (2.3)	38 (4.0)	10 (4.6)	28 (3.8)	25 (4.0)	3 (2.5)
***40**	12 (4.0)	41 (4.3)	13 (6.0)	28 (3.8)	24 (3.9)	4 (3.3)
***41**	4 (1.3)	10 (1.0)	2 (0.9)	8 (1.1)	7 (1.1)	1 (0.8)
***42**	7 (2.3)	17 (1.8)	6 (2.8)	11 (1.5)	10 (1.6)	1 (0.8)
***44**	26 (8.7)	87 (9.1)	11 (5.0)	76 (10.3)	65 (10.5)	11 (9.2)
***45**	3 (1.0)	13 (1.4)	4 (1.8)	9 (1.2)	6 (1.0)	3 (2.5)

***46**	0 (0.0)	1 (0.1)	0 (0.0)	1 (0.1)	1 (0.2)	0 (0.0)
***48**	0 (0.0)	4 (0.4)	1 (0.5)	3 (0.4)	3 (0.5)	0 (0.0)
***49**	12 (4.0)	32 (3.3)	6 (2,8)	26 (3.5)	22 (3.6)	4 (3.3)
***50**	9 (3.0)	22 (2.3)	7 (3.2)	15 (2.0)	13 (2.1)	2 (1.7)
***51**	20 (6.7)	71 (7.4)	12 (5.5)	59 (8.0)	46 (7.4)	13 (10.8)
***52**	6 (2.0)	17 (1.8)	3 (1.4)	14 (1.9)	10 (1.6)	4 (3.3)
***53**	7 (2.3)	25 (2.6)	5 (2.3)	20 (2.7)	19 (3.1)	1 (0.8)
***55**	7 (2.3)	9 (0.9)	2 (0.9)	7 (0.9)	7 (1.1)	0 (0.0)
***56**	1 (0.3)	3 (0.3)	0 (0.0)	3 (0.4)	3 (0.5)	0 (0.0)
***57**	12 (4.0)	19 (2.0)	5 (2.3)	14 (1.9)	14 (2.3)	0 (0.0)
***58**	5 (1.7)	31 (3.2)	7 (3.2)	24 (3.3)	20 (3.2)	4 (3.3)
***73**	1 (0.3)	0 (0.0)	0 (0.0)	0 (0.0)	0 (0.0)	0 (0.0)
***81**	1 (0.3)	5 (0.5)	0 (0.0)	5 (0.7)	5 (0.8)	0 (0.0)
***82**	0 (0.0)	2 (0.2)	0 (0.0)	2 (0.3)	2 (0.3)	0 (0.0)
HLA-C						
***01**	1 (2.7)	37 (3.9)	4 (1.8)	33 (4.5)	29 (4.7)	4 (3.3)
***02**	19 (6.3)	41 (4.3)	10 (4.6)	31 (4.2)	27 (4.4)	4 (3.3)
***03**	24 (8.0)	89 (9.3)	26 (11.9)	63 (8.5)	58 (9.4)	5 (4.2)
***04**	53 (17.7)	179 (18.7)	43 (19.7)	136 (18.4)	127 (20.6)	9 (7.5)
***05**	17 (5.7)	44 (4.6)	12 (5.5)	32 (4.3)	25 (4.0)	7 (5.8)
***06**	28 (9.3)	90 (9.4)	21 (9.6)	69 (9.3)	53 (8.6)	16 (13.3)
***07**	66 (22.0)	204 (21.3)	56 (25.7)	148 (20.1)	114 (18.4)	34 (28.3)
***08**	10 (3.3)	62 (6.5)	9 (4.1)	53 (7.2)	43 (7.0)	10 (8.3)
***12**	27 (9.0)	59 (6.2)	9 (4.1)	50 (6.8)	46 (7.4)	4 (3.3)
***14**	9 (3.0)	18 (1.9)	3 (1.4)	15 (2.0)	15 (2.4)	0 (0.0)
***15**	12 (4.0)	46 (4.8)	10 (5.0)	36 (4.9)	28 (4.5)	8 (6.7)
***16**	15 (5.0)	57 (6.0)	9 (4.1)	48 (6.5)	34 (5.5)	14 (11.4)
***17**	10 (3.3)	27 (2.8)	6 (2.8)	21 (2.8)	18 (2.9)	3 (2.5)
***18**	2 (0.7)	2 (0.2)	1 (0.5)	1 (0.1)	1 (0.2)	0 (0.0)

SC: severe/critical; M: moderate/mild.
